# Asymptotic structural properties of quasi-random saturated structures of RNA

**DOI:** 10.1186/1748-7188-8-24

**Published:** 2013-10-25

**Authors:** Peter Clote, Evangelos Kranakis, Danny Krizanc

**Affiliations:** 1 Biology Department, Boston College, Chestnut Hill, MA, 02467, USA; 2 School of Computer Science, Carleton University, K1S 5B6, Ottawa, Ontario, Canada; 3 Department of Mathematics and Computer Science, Wesleyan University, Middletown, CT, 06459, USA

**Keywords:** RNA secondary structure, Kinetic trap, combinatorial analysis, Zipf distribution

## Abstract

**Background:**

RNA folding depends on the distribution of kinetic traps in the landscape of all secondary structures. Kinetic traps in the Nussinov energy model are precisely those secondary structures that are *saturated*, meaning that no base pair can be added without introducing either a pseudoknot or base triple. In previous work, we investigated asymptotic combinatorics of both *random* saturated structures and of *quasi-random* saturated structures, where the latter are constructed by a natural stochastic process.

**Results:**

We prove that for quasi-random saturated structures with the *uniform distribution*, the asymptotic expected number of external loops is *O*(log*n*) and the asymptotic expected maximum stem length is *O*(log*n*), while under the *Zipf distribution*, the asymptotic expected number of external loops is *O*(log2*n*) and the asymptotic expected maximum stem length is *O*(log*n*/log log*n*).

**Conclusions:**

Quasi-random saturated structures are generated by a stochastic greedy method, which is simple to implement. Structural features of random saturated structures appear to resemble those of quasi-random saturated structures, and the latter appear to constitute a class for which both the generation of sampled structures as well as a combinatorial investigation of structural features may be simpler to undertake.

## Background

RNA is an important biomolecule, now known to play both an *information carrying* role, as in retroviruses, such as HIV, whose genome consists of RNA, as well as a *catalytic* role, as in the the peptidyl transferase catalysis by RNA, which concatenates an amino acid to a growing peptide chain in the formation of a protein on the ribosome [1]. It has recently emerged that RNA plays a wide range of previously unsuspected roles in many biological processes, including *retranslation* of the genetic code (selenocysteine insertion [2], ribosomal frameshift [3]), transcriptional and translational gene regulation [4,5], temperature sensitive conformational switches [6,7], chemical modification of specific nucleotides in the ribosome [8], regulation of alternative splicing [9], etc.

The diverse and biologically important functions performed by RNA molecules depend for the most part on RNA tertiary structure, which is known to be constrained by secondary structure, the latter acting as a scaffold for tertiary contact formation [10]. For this reason, much work has focused on RNA secondary structure prediction [11-14] and on the kinetics of RNA folding [15-17]. In [18], Stein and Waterman pioneered work on asymptotic combinatorics of RNA secondary structures, where they developed recurrence relations to count the number of secondary structures. These recurrence relations were later modified by Nussinov and Jacobson [19] and especially by Zuker [20] to compute the minimum free energy secondary structure.

Formally, a secondary structure for a given RNA nucleotide sequence *a*_1_, …, *a*_*n*_ is a set *S* of base pairs (*i*, *j*), such that *(i)* if (*i*, *j*) ∈ *S* then *a*_*i*_, *a*_*j*_ form either a Watson-Crick (AU,UA,CG,GC) or wobble (GU) base pair, *(ii)* if (*i*, *j*) ∈ *S* then *j* - *i* > *θ* = 3 (a steric constraint requiring that there be at least *θ* = 3 unpaired bases between any two paired bases), *(iii)* if (*i*, *j*) ∈ *S* then for all *j*^′^ ≠ *j* and *i*^′^ ≠ *i*, (*i*^′^, *j*) ∉ *S* and (*i*, *j*^′^) ∉ *S* (nonexistence of base triples), *(iv)* if (*i*, *j*) ∈ *S* and (*k*, *ℓ*) ∈ *S*, then it is not the case that *i* < *k* < *j* < *ℓ* (nonexistence of pseudoknots). For the purposes of this paper, following Stein and Waterman [18], we consider the *homopolymer* model of RNA, in which condition *(i)* is dropped, thus entailing that any base can pair with any other base, and we modify condition *(ii)* so that *θ* = 1. With inessential additional complications in the combinatorics, we could handle the situation where *θ* is any fixed positive constant.

For a given RNA sequence, a *saturated secondary structure* is one such that no base pair can be added without introducing either a pseudoknot or base triple; in other words, saturated structures have a *maximal* number of base pairs, while the Nussinov minimum energy structure has a *maximum* number of base pairs. Since the kinetics of RNA structure formation depend on secondary structure energy landscape, and more particularly on the distribution of kinetic traps (saturated structures), in previous work we have designed an algorithm to compute the number of saturated structures [21], determine the asymptotic number of saturated secondary structures [22] and the expected number of base pairs in saturated and quasi-random saturated structures [23].

Secondary structures are conveniently displayed in Vienna *dot bracket notation*, consisting of a balanced parenthesis expression with dots, where an unpaired nucleotide at position *i* is depicted by a dot at that position, while a base pair (*i*, *j*) is depicted by the presence of matching left and right parentheses located respectively at positions *i* and *j*. The minimum free energy secondary structure of the selenocysteine insertion (SECIS) sequence fruA, given by

CCUCGAGGGGAACCCGAAAGGGACCCGAGAGG((((..(((...(((....))).)))..))))

is a saturated structure. In contrast, the following structure for the Gag/pro ribosomal frameshift site of mouse mammary tumor virus [24] is not only not saturated, but includes a pseudoknot, as shown by the square bracket notation necessary to show the crossing base pairs.

AAAAAACUUGUAAAGGGGCAGUCCCCUAGCCCCGCUCAAAAGGGGGAUG..............(((((.[[[[[[[.)))))........]]]]]]].

Turning to the homopolymer model considered in this paper, there are precisely five saturated structures for RNA of length 5

((∙)),∙(∙∙),(∙∙)∙,(∙)∙∙,∙∙(∙)

and there are precisely eight saturated structures for RNA of length 6

$$\begin{array}{lll} & \;(\;\;(\;\;∙\;\;)\;\;)∙,∙(\;\;(\;\;∙\;\;)\;\;),(\;\;(\;\;∙\;\;)∙\;\;),(\;\;∙(\;\;∙\;\;)\;\;),\; & \;\\ & \;(\;\;(\;\;∙∙\;\;)\;\;),(\;\;∙\;\;)(\;\;∙\;\;),(\;\;∙∙\;\;)∙∙,∙∙(\;\;∙∙\;\;).\; & \; \end{array}$$

Having defined *saturated* structure, we now define a stochastic greedy process to generate *random* saturated structures, technically denoted *quasi-random saturated structures*. This notion was defined in [23], where we showed that the expected number of base pairs in quasi-random saturated structures is 0.340633 · *n*, just slightly more than the expected number 0.337361 · *n* of base pairs in all saturated structures.

**Figure 1:**
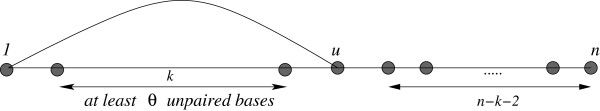
**Base 1 is base-paired by selecting a random base *u *such there are at least *θ *unpaired bases enclosed between 1 and *u*.** By iterating this procedure, we obtain a *greedy stochastic algorithm* to sample *quasi-random* secondary structures.

Consider the following stochastic process to generate a saturated structure. Suppose that *n* bases are arranged in sequential order on a line. Select the base pair (1,*u*) by choosing *u*, where *θ* + 2 ≤ *u* ≤ *n*, at random with probability 1 / (*n* - * θ * - 1). The base pair joining 1 and *u* partitions the line into two parts. The left region has *k* bases strictly between 1 and *u*, where *k* ≥ *θ*, and the right region contains the remaining *n* - *k* - 2 bases properly contained within endpoints *k* + 2 and *n* (see Figure 1). Proceed recursively on each of the two parts. Observe that the secondary structures produced by our stochastic process will always base pair with the leftmost available base, and that the resulting structure is always saturated. Note that the probability *p*_*i*,*j*_ that (*i*, *j*) is a base pair in a saturated structure is *not* the same as the probability *q*_*i*,*j*_ that (*i*,*j*) is a base pair in a quasi-random saturated structure (this was shown in [23], using a program we wrote to generate saturated structures).

## Results and discussion

With these definitions, we are now in a position to state some results concerning *structural features* of (quasi) random saturated structures. Under the *uniform distribution*, we show that the asymptotic expected number of external loops is *O*(log*n*), and the expected maximum stem length is *O*(log*n*). In contrast, under the *Zipf distribution*, the asymptotic expected number of external loops is *O*(log2*n*), and the expected maximum stem length is *O*(log*n*/ log log*n*)^a^.

In the literature on RNA combinatorics ([18] and subsequent papers), combinatorial results have been proved for the homopolymer as well as for the Bernouilli model, in which latter one assumes a *stickiness* parameter *p* = 2(*p*_*A*_*p*_*U*_ + *p*_*G*_*p*_*U*_ + *p*_*G*_*p*_*C*_) that any two positions can base-pair. To the best of our knowledge, the current paper appears to be one of the first combinatorial analyses of RNA secondary structures, which involves the Zipf distribution for base pairs.

## Conclusions

Saturated secondary structures form natural kinetic traps in the energy landscape with respect to the Nussinov energy model [19], in that it is energetically unfavorable to move from a saturated structure to any neighboring structure that differs by one base pair. However, there is currently no program to sample saturated secondary structures with respect to the Nussinov energy (given either a homopolymer or an RNA sequence), although the programs we developed in [21,22] could be extended to do so for both homopolymers and RNA sequences. (Note that the program RNAsat, described in [25], can sample saturated structures in the Turner energy landscape, and the program RNAlocopt, described in [26], can sample *locally optimal* structures in the Turner energy landscape). In contrast, it is extremely simple to implement a program to sample quasi-random saturated structures, thus permitting one to easily obtain an idea of various structural features in the ensemble of quasi-random structures. We expect many structural features to be approximately shared between the random saturated structures and quasi-random saturated structures – for instance, as earlier mentioned, the expected number of base pairs in quasi-random saturated structures is 0.340633·*n*, while the expected number of base pairs in saturated structures is 0.337361·*n*, almost the same value [23].

Generally, it requires substantial effort involving the application of deep results from complex analysis, such as the Flajolet-Odlyzko theorem [27] or the Drmota-Lalley-Woods theorem [28-30] (see also the text by Flajolet and Sedgewick [31]) to prove asymptotic results, such as the fact that the asymptotic number of saturated structures is 1.07427 · *n*^-3/2^ · 2.35467^*n*^ and the asymptotic expected number of base pairs is 0.337361 · *n*, and the asymptotic expected number of hairpins is 0.323954 · 1.69562^*n*^[23]. In contrast, the argument given in this paper is elementary, not requiring complex analysis. Taken together we believe that the stochastic greedy method, described in Figure 1, performs reasonably well in sampling saturated structures, that appear to be representative of the ensemble of all saturated structures, and supports a combinatorial analysis that may be simpler than that required for all saturated structures.

## Methods

### Structural properties of quasi-random saturated secondary structures

**Figure 2:**
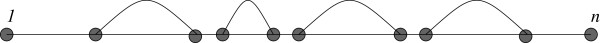
A sequence of external base pairs.

**Figure 3:**
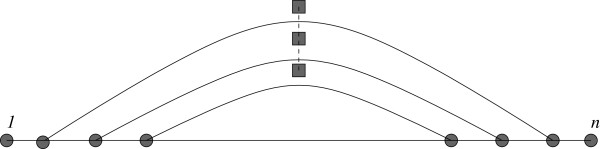
A sequence of nested base pairs.

Given secondary structure *S*, an *external base pair* is a base pair (*i*, *j*) ∈ *S*, which is not interior to any other base pair of *S*; i.e. there is no (*x*, *y*) ∈ *S* with the property that *x* < *i* < *j* < *y*. A sequence of external base pairs is a sequence (*a*_*i*_, *b*_*i*_), *i* = 1, 2, …, *k* such that *a*_*i*_ < *b*_*i*_ < *a*_*i*+1_ < *b*_*i*+1_, for all *i* < *k*, and for which each (*a*_*i*_, *b*_*i*_) is external. The base pairs (*a*_*i*_, *b*_*i*_) are said to *close* the corresponding *external loops*; see Figure 2. The *number of external loops* of a given secondary structure *S* is defined to be the total number of external base pairs in *S*. We define a *stem* of length *k* to be a sequence of nested base pairs (see Figure 3) (*a*_*i*_, *b*_*i*_), *i* = 1, 2, …, *k*, such that *a*_*i*_ < *a*_*i*+1_ < *b*_*i*+1_ < *b*_*i*_, for all *i* < *k*. The *stem length* of a given secondary structure *S* is defined here to be the maximum length of all stems in *S*; i.e. the maximum number of nested base pairs in *S*.

**Figure 4:**
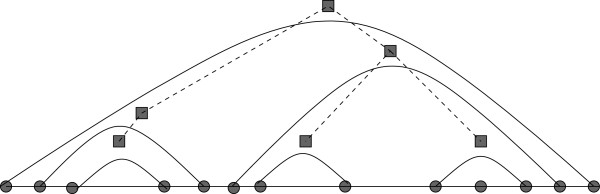
The tree associated with the given set of base pairs.

Our study of structural properties of random saturated secondary structures is facilitated by defining a graph that resembles the graph on page 333 of [32]; however, note that the formal definition is slightly different than that of [32]. Given a secondary structure *S* on the nucleotide sequence [1, *n*], define the associated graph *G*(*S*) = (*V*, *E*), whose vertex set *V* consists of base pairs *v* = (*i*, *j*) in *S*, and whose undirected edge set *E* consists of pairs {*v*, *v*^′^} of nested vertices, *v* = (*i*, *j*) and *v*^′^ = (*i*^′^, *j*^′^), that can directly *see* each other; i.e. {*v*, *v*^′^} ∈ *E* exactly when *i* < *i*^′^ < *j*^′^<*j* and there does not exist a base pair (*x*, *y*) ∈ *S*, such that *i* < *x* < *i*^′^ < *j*^′^ < *y* < *j*, or vice-versa with the roles of *v*, *v*^′^ reversed. Figure 4 depicts the graph *G*(*S*) associated with the saturated secondary structure *S*.

In general *G*(*S*) is a forest; i.e., a set of trees. In the sequel we determine the size of several structural parameters of random saturated secondary structures, in particular, expected stem length and expected number of external loops. These parameters are studied both for the uniform and Zipf distributions. Before proceeding any further, we first define the probability distributions to be considered.

#### Probability distributions

*Zipf’s law* is the observation first made by the deceased Harvard linguist, George Kingsley Zipf, that the frequency *p*_*i*_ of English words, when graphed against their rank *i* (in the list of English words sorted in decreasing order with respect to frequency), obeys the power law *p*_*i*_ ≈ *i*^ - *α*^. More generally, Zipf’s law is the statement of a power law, when plotting frequency against rank (Zipf’s first law) or when plotting frequency against reverse rank (Zipf’s second law). In bioinformatics, Zipf’s law has been observed in the frequency/rank plot of differentially expressed gene in microarray data [33], as well as in the frequency/rank plot for protein structures [34], where there are a few very frequent structures, and very many rare structures. In the remainder of the paper, we consider probability distributions related to Zipf’s law.

A node, say 1 ≤ *u* ≤ *n*, is chosen at random with the *α-Zipf* distribution, if the probability that a given base pair (1,*u*) is chosen is equal to $\frac{1}{{\left(u-1\right)}^{\alpha }H_{\alpha }(n-1)}$, where

$$H_{\alpha }(n-1)=\overset{n-1}{\underset{k=1}{\sum }}\frac{1}{k^{\alpha }}$$

is defined to be the *α*-harmonic number of *n* - 1. The expected number of base pairs for arbitrary threshold *θ* is denoted by $E_{n}^{\theta }$, for random saturated secondary structures on *n* bases, generated by the *α*-Zipf stochastic process. $E_{n}^{0}$ satisfies the following recursive formula

(1) $$\begin{array}{l} E_{n}^{0}=1+\frac{1}{H_{\alpha }(n-1)}\overset{n-2}{\underset{k=0}{\sum }}\frac{1}{{\left(k+1\right)}^{\alpha }}(E_{k}^{0}+E_{n-k-2}^{0}), \end{array}$$

for all *n* ≥ 2.

Observe that when *α* = 0 the *α*-Zipf distribution is the same as the uniform distribution, while if *α* = 1, we have the (classical) Zipf distribution [35]. Moreover, observe that as *α* increases, “shorter” base pairs are being selected with higher probability by the stochastic process described in equation (1).

The stochastic process of generating random saturated secondary structures, according to equation (1), is of the “divide-and-conquer” type, very common in computer science, where well-known algorithms such as QUICKSORT choose a division point according to the uniform distribution. Stochastic algorithms of this kind have been intensively studied for the uniform distribution. Known results suggest that the probability distribution for the number of base pairs in random saturated structures, generated by the earlier described stochastic process (uniform choice of base pairs) is asymptotically Gaussian (see [36] and [37]). We also note that structural features of trees have been well studied including the expected depth and the exact distribution of the depth; see, for instance, [36,38,39]. In the sequel, we consider a random binary search tree with *n* nodes obtained by inserting *n* i.i.d. random variables *X*_1_, …, *X*_*n*_. Careful analysis of [36] and [39] implies our results in the section on the uniform distribution. However we will use a different and simpler technique that enables the analysis not only for the uniform distribution in the following section concerning the Uniform Distribution, but also for the Zipf distribution in the section following this section.

An important observation concerns the threshold *θ* considered above. All the results proved in this section are “upper bounds” and therefore it is easily seen that they are valid for any threshold *θ* ≥ 0. Therefore to simplify proofs in the sequel we consider the case of threshold *θ* = 0.

### Uniform distribution

The main theorem of this section concerns stem length and number of external loops of random saturated structures *S*, generated by a natural stochastic process associated with the tree graph *G*(*S*). Throughout the remainder of the paper, we state results in terms of *random saturated* structures, although we intend to mean only those structures generated by the stochastic process associated with the graph *G*(*S*); we will distinguish between the uniform and *α*-Zipf variant of the stochastic process. Without this convention, statements of lemmas and theorems would be too cumbersome.

#### 

**Theorem 1.** With high probability, the number of external loops and the maximum stem length of random saturated structures generated by the uniform distribution variant is *O*(log*n*).

*Proof.* Before we give the proof of the main theorem it will be necessary to give the proof of two lemmas. In the first lemma we consider the expected number of external loops.

#### 

**Lemma 1.** With high probability, the number of external loops is *O*(log*n*).

*Proof.* We define a sequence of random variables *X*_1_, *X*_2_, …, *X*_*t*_ by induction as follows. Let *X*_1_ be the random variable selecting a base *k* chosen among 2, 3, …, *n* randomly and independently with the uniform distribution in order to form a base pair (1,*k*). By induction, assume that *X*_1_, …, *X*_*t*_ have been defined. Let *X*_*t*+1_ be the random variable selecting a base *k* chosen among *X*_*t*_ + 2,*X*_*t*_ + 3, …, *n* randomly and independently with the uniform distribution in order to form a base pair (*X*_*t*_ + 1, *k*). Next we estimate bounds on $E[X_{t}]$, for all *t*. Indeed, observe that $P[X_{1}=k]=\frac{1}{n-1}$ and

$$\begin{array}{llll} E[\;X_{1}] & = & \overset{n}{\underset{i=2}{\sum }}i\cdot \frac{1}{n-1} & \\ & = & \frac{1}{n-1}\overset{n}{\underset{i=2}{\sum }}i & \\ & = & \frac{1}{n-1}\left(\frac{n(n+1)}{2}-1\right). & \end{array}$$

Next we compute the conditional probability

$$\begin{array}{llll} E[\;X_{t+1}|X_{t}=k] & = & \overset{n}{\underset{i=k+2}{\sum }}i\cdot P[X_{t+1}=i|X_{t}=k] & \\ & = & \overset{n-1}{\underset{i=k+2}{\sum }}i\cdot \frac{1}{n-k-1} & \\ & = & \frac{1}{n-k-1}\overset{n-1}{\underset{i=k+2}{\sum }}i & \\ & = & \frac{1}{n-k-1}\left(\overset{n-1}{\underset{i=0}{\sum }}i-\overset{k+1}{\underset{i=0}{\sum }}i\right) & \\ & = & \frac{n+k+1}{2}-\frac{n+k+1}{2(n-k-1)} & \\ & \geq & \frac{n+k+1}{4}, & \end{array}$$

where the last inequality is valid for *k* + 3 ≤ *n*.

Finally, we can estimate

$$\begin{array}{llll} E[\;X_{t+1}] & = & E[E[\;X_{t+1}|X_{t}]] & \\ & = & \underset{k}{\sum }E[\;X_{t+1}|X_{t}=k]\cdot P[X_{t}=k] & \\ & \geq & \underset{k}{\sum }\frac{n+k+1}{4}\cdot P[X_{t}=k] & \\ & = & \frac{n+1}{4}+\frac{1}{4}\underset{k}{\sum }k\cdot P[X_{t}=k] & \\ & = & \frac{n+1}{4}+\frac{1}{4}E[X_{t}] & \\ & = & \frac{n+1}{4}\cdot \left(1+2^{-1}+\cdots +2^{-t}\right) & \\ & = & \frac{n+1}{2}\cdot \left(1-2^{-t-1}\right). & \end{array}$$

We are interested in determining the behavior of the random variable, whose value is the number of external loops in random saturated structures.

(2) $$T_{n}=\text{min}\{t:X_{t+1}\geq (n+1)/2\}.$$

From this we derive

$$\begin{array}{llll} P[\;T_{n}>t] & = & P[X_{t+1}<(n+1)/2] & \\ & = & P[(n+1)/2-X_{t+1}>0] & \\ & \leq & E[(n+1)/2-X_{t+1}] & \\ & = & \frac{n+1}{2}-E[X_{t+1}] & \\ & \leq & \frac{n+1}{2}-\frac{n+1}{2}\cdot \left(1-2^{-t-1}\right) & \\ & = & \frac{n+1}{2}\cdot 2^{-t-1}. & \end{array}$$

In particular, $P[\;T_{n}>(1+\epsilon )\log n)]\leq n^{-\epsilon }+o(n^{-\epsilon })$. This completes the proof of Lemma 1. □

Next we prove the following lemma.

#### 

**Lemma 2.** With high probability, the maximum stem length is *O*(log*n*).

*Proof.* According to the recursive construction, at each stage after a base pair is chosen at random in the subsequent stages, base pairs are nested within this base pair. Therefore, the maximum stem length equals the maximum number of nested base pairs. This latter number can also be obtained as follows. We define the following sequence *Y*_1_, *Y*_2_, …, *Y*_*t*_ of random variables. A base is chosen among 2, 3, …, *n* randomly and independently with the uniform distribution. Let *Y*_1_ be the resulting random variable. By induction, assume that *Y*_1_, …, *Y*_*t*_ have been defined. To define the random variable *Y*_*t*+1_, a base is chosen among *t* + 2, …, *Y*_*t*_ - 1 randomly and independently with the uniform distribution. Clearly, this procedure halts when *Y*_*t*_ ≤ *t* + 2 and it follows that the maximum number of nested base pairs is also the number *t* of iterations before halting. Therefore we are interested in knowing the behavior of the random variable

(3) $$T^{\prime }=\text{min}\{t:Y_{t}\leq t+2\}$$

(notice the dependence of the random variable *T*^′^ on *n*).

Observe that since by definition *Y*_*i*+1_ is chosen among *i* + 2, *i* + 3, …, *Y*_*i*_ - 1 randomly and independently with the uniform distribution, for any integer *k* ≥ *i* + 2, $E[\;Y_{i+1}|Y_{i}=k]=\frac{k+i+1}{2}.$ Consider the random variable $E[\;Y_{i+1}|Y_{i}]$ whose value at *k* is equal to $E[Y_{i+1}|Y_{i}=k]$. Using well-known identities on conditional probabilities, we can derive the following equalities.

$$\begin{array}{llll} E[\;Y_{i+1}] & = & E[\;E[\;Y_{i+1}|Y_{i}]\;] & \\ & = & \underset{k}{\sum }E[\;Y_{i+1}|Y_{i}=k]\cdot P[\;Y_{i}=k] & \\ & = & \underset{k}{\sum }\frac{k+i+1}{2}\cdot P[\;Y_{i}=k] & \\ & = & \frac{1}{2}\underset{k}{\sum }k\cdot P[\;Y_{i}=k]+\frac{i+1}{2} & \\ & = & \frac{1}{2}E[\;Y_{i}]+\frac{i+1}{2}. & \end{array}$$

In particular, since $E[\;Y_{1}]=\frac{n+2}{2}$, we conclude that $E[\;Y_{t}]\leq {\left(1/2\right)}^{t}\cdot n$. Finally, we can derive $P[\;T^{\prime }>t]=P[\;Y_{t}>0]\leq E[\;Y_{t}]\leq {\left(1/2\right)}^{t}\cdot \mathrm{n.}$ It follows that $P[\;T^{\prime }>(1+\epsilon )\log n)]\leq n^{-\epsilon }.$

We are not yet completely done with the proof of Lemma 2. The proof shows that with high probability, the leftmost sequence of base pairs given by the recursive construction has length at most *O*(log*n*). We would like to prove the same for any sequence of nested base pairs. To this effect, define random intervals *I*_*s*_, where *s* is a finite sequence of 0s and 1s, by induction on the length of *s*. Consider the interval *I*_*∅*_ = [1, *n*]. Assuming that *I*_*s*_ = [*a*_*s*_, *b*_*s*_] has already been defined, we consider a random process that splits it at random into two subintervals, i.e., choose an integer *r* ∈ *I*_*s*_ randomly and independently with the uniform distribution and let *I*_*s*0_ = [*a*_*s*_, *r*] and *I*_*s*1_ = [*r* + 1, *b*_*s*_]. Since $E[\;|I_{\text{sb}}|]\leq \frac{1}{2}\cdot E[\;|I_{s}|]$ it follows that the expected length of *I*_*s*_ is at most 2^-|*s*|^. Now consider the random variable *T*^′′^ which is defined as follows *T*^′′^ = min{*k*:∃*s*(|*s*| = *k* & *I*_*s*_ = *∅*)}(notice the dependence of the random variable *T*^′′^ on *n*) and observe that *T*^′′^ > *k* if and only if ∀*s*(|*s*| = *k* ⇒ *I*_*s*_ ≠ *∅*). Therefore

$$\begin{array}{llll} P[\;T^{\prime \prime }>k]\; & = & \;P[\underset{k:|s|=k}{\text{min}}|I_{s}|>0]\leq E[\underset{k:|s|=k}{\text{min}}|I_{s}|] & \\ & \leq & \;E[\;|I_{s}|],(\text{for all sequences}\;s\;\text{such that}\;|s|=k) & \\ & \leq & \;2^{-k}. & \end{array}$$

As a consequence we conclude that $P[T^{\prime \prime }>(1+\epsilon )\log n)]\leq n^{-\epsilon }.$ This completes the proof of Lemma 2. □

Finally, we can complete the proof of the main result of Theorem 1 since this is now immediate from Lemmas 1 and 2.

### Zipf distribution

It is possible to consider other probability distributions like Zipf and generalized *a*-Zipf. The Zipf distribution (first considered in [35]) is perhaps the most interesting because it favors base pairs at a shorter distance. A base pair (1, *u*), is chosen at random with the Zipf distribution. I.e., the probability that the base pair (1, *u*) is selected is equal to $\frac{1}{\left(u-1)H(n-1\right)}$, where

$$H(n-1)=\overset{n-1}{\underset{k=1}{\sum }}\frac{1}{k}$$

is defined to be the (*n* - 1)st harmonic number. As before, the chord joining 1 and *u* partitions the ring into two parts. One part has *k* bases between 1 and *u*, where *k* ≤ *n* - 2, and the other part has the remaining *n*-*k*-2 bases (see Figure 1).

Define *Z*_*n*_ to be the expected number of base pairs of a random saturated secondary structure with *n* bases, where *n* ≥ 2. A base pair (1, *u*) is added as follows. Select *u* ≥ 2 at random among 2, 3, …, *n* with probability $\frac{1}{\left(u-1)H(n-1\right)}$.

This gives rise to the following formula

(4) $$Z_{n}=1+\frac{1}{H(n-1)}\overset{n-2}{\underset{k=0}{\sum }}\frac{1}{k+1}(Z_{k}+Z_{n-k-2}),$$

for all *n* ≥ 2. The main theorem of this section concerns the overall structure of random secondary structures.

#### 

**Theorem 2.** With high probability, random saturated secondary structures generated by the Zipf distribution have *O*(log2*n*) external loops and stem length *O*(log*n*/ log log*n*).

*Proof.* Before we give the proof, it will be necessary to give the proof of two lemmas. In the first lemma we look at the number of external loops.

#### 

**Lemma 3.** With high probability, the number of external loops is *O*(log2*n*).

*Proof.* We define a sequence of random variables *X*_1_, *X*_2_, …, *X*_*t*_ by induction as follows. Let *X*_1_ be the random variable resulting when the base pair (1, *k*) is formed by a selecting a base *k* among 2, 3, …, *n* randomly and independently with the Zipf distribution. By induction, assume that *X*_1_, …, *X*_*t*_ have been defined. Let *X*_*t*+1_ be the random variable resulting when the base pair (*X*_*t*_ + 1, *k*) is formed by selecting a base *k* is chosen among *X*_*t*_ + 1, *X*_*t*_ + 2, …, *n* randomly and independently with the Zipf distribution. Next we compute $E[X_{t}]$, for all *t*. Indeed, observe that $P[\;X_{1}=k]=\frac{1}{\left(k-1)H(n-1\right)}$ and

$$\begin{array}{llll} E[\;X_{1}] & = & \overset{n}{\underset{i=2}{\sum }}i\cdot \frac{1}{\left(i-1)H(n-1\right)} & \\ & = & \frac{n-1}{H(n-1)}+1. & \end{array}$$

Next we compute the conditional probability

$$\begin{array}{lll} \;E[\;X_{t+1}|X_{t}=k]\; & =\;\overset{n}{\underset{i=k+1}{\sum }}i\cdot P[\;X_{t+1}=i|X_{t}=k]\; & \;\\ \; & =\;\overset{n}{\underset{i=k+1}{\sum }}i\cdot \frac{1}{\left(i-k-1)H(n-k-1\right)}\; & \;\\ \; & =\;\frac{1}{H(n-k-1)}\overset{n}{\underset{i=k+1}{\sum }}\frac{i}{i-k-1}\; & \;\\ \; & =\;\frac{1}{H(n-k-1)}\overset{n}{\underset{i=k+1}{\sum }}\left(\frac{i-k-1}{i-k-1}\right)\; & \;\\ & \;\;\;+\left(\frac{k+1}{i-k-1}\right)\; & \;\\ \; & =\;\frac{n-k-1}{H(n-k-1)}+(k+1).\; & \; \end{array}$$

Finally, we can calculate

$$\begin{array}{llll} E[\;X_{t+1}] & = & E[\;E[\;X_{t+1}|X_{t}]] & \\ & = & \underset{k}{\sum }E[\;E[\;X_{t+1}|X_{t}=k]]\cdot P[\;X_{t}=k] & \\ & = & \underset{k}{\sum }\left((k+1)+\frac{n-k-1}{H(n-k-1)}\right)\cdot P[\;X_{t}=k] & \\ & = & 1+E[\;X_{t}]+\underset{k}{\sum }\frac{n-k-1}{H(n-k-1)}\cdot P[\;X_{t}=k] & \\ & \geq & 1+E[\;X_{t}]+\frac{1}{H(n-1)}\underset{k}{\sum }(n-k-1)\cdot P[\;X_{t}=k] & \\ & = & 1+E[\;X_{t}]+\frac{1}{H(n-1)}\left(n-1-E[\;X_{t}]\right) & \\ & \geq & \frac{n-1}{H(n-1)}+\left(1-\frac{1}{H(n-1)}\right)E[\;X_{t}]. & \end{array}$$

Elementary calculations using this last inequality show that

$$E[\;X_{t+1}]\geq (n-1)\left(1-{\left(1-\frac{1}{H(n-1)}\right)}^{t+2}\right).$$

We are interested in determining the behavior of the random variable, whose value is the number of external loops; i.e. the size of the largest sequence of external base pairs. Define the random variable

(5) $$T_{n}=\text{min}\{t:X_{t+1}\geq n-1\}.$$

From this we derive

$$\begin{array}{llll} P[\;T_{n}>t] & = & P[X_{t+1}<n-1] & \\ & = & P[n-1-X_{t+1}>0] & \\ & \leq & E[n-1-X_{t+1}] & \\ & = & n-1-E[X_{t+1}] & \\ & \leq & n-1-(n-1)\left(1-{\left(1-\frac{1}{H(n-1)}\right)}^{t+2}\right) & \\ & = & (n-1){\left(1-\frac{1}{H(n-1)}\right)}^{t+2}. & \end{array}$$

In particular, since *H*(*n* - 1) ∼ ln*n* we conclude that $P[\;T_{n}>\epsilon \overset{2}{\ln}n)]\leq n^{-\epsilon }.$ This completes the proof of Lemma 3. □

The next result concerns the maximum stem length. We can prove the following result.

#### 

**Lemma 4.** With high probability, the maximum stem length is *O*(log*n*/ log log*n*).

*Proof.* According to the recursive construction, at each stage after a base pair is chosen at random in the subsequent stages base pairs are nested within this base pair. Therefore, the maximum stem length is equal to the maximum number of nested base pairs. This latter number can also be obtained, by investigating a sequence of random variables *Y*_1_, *Y*_2_, …, *Y*_*t*_, defined as follows. Choose a base among 2, 3, …, *n* - 1 randomly and independently with the Zipf distribution. Let *Y*_1_ be the resulting random variable. By induction, assume that *Y*_1_, …, *Y*_*t*_ have been defined. To define the random variable *Y*_*t*+1_, a base is chosen among *t* + 2, *t* + 3, …, *Y*_*t*_ - 1 randomly and independently with the Zipf distribution. Clearly, this procedure halts when *Y*_*t*_ = 1 and it follows that the maximum number of nested base pairs is also the number *t* of iterations before halting. Therefore we are interested to know the behavior of the random variable

(6) $$T^{\prime }=\text{min}\{t:Y_{t}>0\}$$

(notice the dependence of the random variable *T*^′^ on *n*).

Observe that since by definition *Y*_*i*+1_ is chosen among *i* + 2, *i* + 3, …, *Y*_*i*_ - 1 randomly and independently with the Zipf distribution, for any integer *k* ≥ *i* + 2,

$$E[\;Y_{i+1}|Y_{i}=k]=\frac{k-i-1}{H(k-i-1)}.$$

Consider the random variable $E[\;Y_{i+1}|Y_{i}]$ whose value at *k* is equal to $E[\;Y_{i+1}|Y_{i}=k]$. Using well-known identities on conditional probabilities we can derive the following inequalities.

$$\begin{array}{llll} E[\;Y_{i+1}] & = & E[\;E[\;Y_{i+1}|Y_{i}]\;] & \\ & = & \underset{k}{\sum }E[\;E[\;Y_{i+1}|Y_{i}=k]]\cdot P[\;Y_{i}=k] & \\ & = & \underset{k\geq i+2}{\sum }\frac{k-i-1}{H(k-i-1)}\cdot P[\;Y_{i}=k] & \\ & \leq & \underset{k\geq i+2}{\sum }\frac{k}{H(k)}\cdot P[\;Y_{i}=k] & \\ & \leq & \frac{1}{H(i+2)}E[\;Y_{i}], & \end{array}$$

where we used the fact that the fraction *n* / *H*(*n*) is monotone increasing in *n*. In particular, since $E[\;Y_{1}]=\frac{n-2}{H(n-2)}$, we conclude that $E[\;Y_{t}]\leq \frac{n-2}{H(t+1)\cdot H(t)\cdots H(2)}$. Finally, we can derive

$$\begin{array}{llll} P[\;T^{\prime }>t] & = & P[\;Y_{t}>0] & \\ & \leq & E[\;Y_{t}] & \\ & \leq & \frac{n-2}{H(t+1)\cdot H(t)\cdots H(2)} & \\ & \leq & \frac{n-2}{H{\left(t/2\right)}^{t/2}}. & \end{array}$$

In particular,

$$P\left[T^{\prime }>(1+\epsilon )\frac{\log n}{\ln\ln n}\right]\leq n^{-\epsilon }.$$

The proof shows that the leftmost sequence of base pairs given by the recursive construction of the random secondary structure has length at most *O*(log*n*/ log log*n*) with high probability. We would like to prove the same for any sequence of nested base pairs. It is easily seen that a proof similar to the one presented above works. This completes the proof of Lemma 4. □

If we now combine Lemmas 3 and 4 we derive the proof of Theorem 2.

## Endnote

^a^ Throughout this paper all logarithms are in base 2.

## Competing interests

The authors declare that they have no competing interests.

## Authors’ contributions

All three authors developed the results and wrote the paper. All authors read and approved the final manuscript.

## References

[B1] Weinger JS Parnell KM Dorner S Green R Strobel SA Substrate-assisted catalysis of peptide bond formation by the ribosome Nat Struct Mol Biol 2004 11 1101 1106 10.1038/nsmb841 15475967

[B2] Böck A Forschhammer K Heider J Baron C Selenoprotein synthesis: An expansion of the genetic code Trends Biochem Sci 1991 16 463 467 1838215 10.1016/0968-0004(91)90180-4

[B3] Bekaert M Bidou L Denise A Duchateau-Nguyen G Forest J Froidevaux C Hatin I Rousset J Termier M Towards a computational model for -1 eukaryotic frameshifting sites Bioinformatics 2003 19 327 335 10.1093/bioinformatics/btf868 12584117 PMC7109833

[B4] Lim L Glasner M Yekta S Burge C Bartel D Vertebrate microRNA genes Science 2003 299 5612 1540 10.1126/science.1080372 12624257

[B5] Mandal M Boese B Barrick J Winkler W Breaker R Riboswitches control fundamental biochemical pathways in Bacillus subtilis and other bacteria Cell 2003 113 5 577 586 10.1016/S0092-8674(03)00391-X 12787499

[B6] Chowdhury S Ragaz C Kreuger E Narberhaus F Temperature-controlled structural alterations of an RNA thermometer J Biol Chem 2003 278 48 47915 47921 10.1074/jbc.M306874200 12963744

[B7] Tucker BJ Breaker RR Riboswitches as versatile gene control elements Curr Opin Struct Biol 2005 15 3 342 348 10.1016/j.sbi.2005.05.003 15919195

[B8] Omer A Lowe T Russell A Ebhardt H Eddy S Dennis P Homologues of small nucleolar RNAs in Archaea Science 2000 288 517 522 10.1126/science.288.5465.517 10775111

[B9] Cheah MT Wachter A Sudarsan N Breaker RR Control of alternative RNA splicing and gene expression by eukaryotic riboswitches Nature 2007 447 7143 497 500 10.1038/nature05769 17468745

[B10] Banerjee A Jaeger J Turner D Thermal unfolding of a group I ribozyme: The low-temperature transition is primarily disruption of tertiary structure Biochemistry 1993 32 153 163 10.1021/bi00052a021 8418835

[B11] Zuker M Mfold web server for nucleic acid folding and hybridization prediction Nucleic Acids Res 2003 31 13 3406 3415 10.1093/nar/gkg595 12824337 PMC169194

[B12] Knudsen B Hein J Pfold: RNA secondary structure prediction using stochastic context-free grammars Nucleic Acids Res 2003 31 13 3423 3428 10.1093/nar/gkg614 12824339 PMC169020

[B13] Hofacker I Vienna RNA secondary structure server Nucleic Acids Res 2003 31 3429 3431 10.1093/nar/gkg599 12824340 PMC169005

[B14] Markham NR Zuker M UNAFold: software for nucleic acid folding and hybridization Methods Mol Biol 2008 453 3 31 10.1007/978-1-60327-429-6_1 18712296

[B15] Flamm C Fontana W Hofacker IL Schuster P RNA folding at elementary step resolution RNA 2000 6 325 338 10.1017/S1355838200992161 10744018 PMC1369916

[B16] Xayaphoummine A Bucher T Isambert H Kinefold web server for RNA/DNA folding path and structure prediction including pseudoknots and knots Nucleic Acids Res 2005 33 Web W605 W610 15980546 10.1093/nar/gki447 PMC1160208

[B17] Danilova LV Pervouchine DD Favorov AV Mironov AA RNAKinetics: a web server that models secondary structure kinetics of an elongating RNA J Bioinform Comput Biol 2006 4 2 589 596 10.1142/S0219720006001904 16819804

[B18] Stein PR Waterman MS On some new sequences generalizing the Catalan and Motzkin numbers Discrete Math 1978 26 261 272

[B19] Nussinov R Jacobson AB Fast algorithm for predicting the secondary structure of single stranded RNA Proc Natl Acad Sci U S A 1980 77 11 6309 6313 10.1073/pnas.77.11.6309 6161375 PMC350273

[B20] Zuker M Stiegler P Optimal computer folding of large RNA sequences using thermodynamics and auxiliary information Nucleic Acids Res 1981 9 133 148 10.1093/nar/9.1.133 6163133 PMC326673

[B21] Clote P An efficient algorithm to compute the landscape of locally optimal RNA secondary structures with respect to the Nussinov-Jacobson energy model J Comput Biol 2005 12 83 101 10.1089/cmb.2005.12.83 15725735

[B22] Clote P Combinatorics of saturated secondary structures of RNA J Comput Biol 2006 13 9 1640 1657 10.1089/cmb.2006.13.1640 17147486

[B23] Clote P Kranakis E Krizanc D Salvy B Asymptotics of canonical and saturated RNA secondary structures J Bioinform Comput Biol 2009 7 5 869 893 10.1142/S0219720009004333 19785050

[B24] Van Batenburg FH Gultyaev AP Pleij CW PseudoBase: structural information on RNA pseudoknots Nucleic Acids Res 2001 29 194 195 10.1093/nar/29.1.194 11125088 PMC29770

[B25] Waldispuhl J Clote P Computing the partition function and sampling for saturated secondary structures of RNA, with respect to the Turner energy model J Comput Biol 2007 14 2 190 215 10.1089/cmb.2006.0012 17456015

[B26] Lorenz WA Clote P Computing the partition function for kinetically trapped RNA secondary structures PLoS One 2011 6 e16178 10.1371/journal.pone.0016178 21297972 PMC3030561

[B27] Flajolet P Odlyzko A Singularity analysis of generating functions SIAM J Discrete Math 1990 3 2 216 240 10.1137/0403019

[B28] Drmota M Systems of functional equations Random Struct Alg 1997 10 103 124 10.1002/(SICI)1098-2418(199701/03)10:1/2<103::AID-RSA5>3.3.CO;2-0

[B29] Lalley SP Finite range random walk on free groups and homogeneous trees Ann Probab 1993 21 2087 2130 10.1214/aop/1176989012

[B30] Woods AR Coloring rules for finite trees, and probabilities of monadic second order sentences Random Struct Alg 1997 10 453 485 10.1002/(SICI)1098-2418(199707)10:4<453::AID-RSA3>3.0.CO;2-T

[B31] Sedgewick R Flajolet P Analytic Combinatorics 2009 Cambridge: Cambridge University [ISBN-13: 9780521898065]

[B32] Waterman MS Introduction to Computational Biology 1995 Boca Raton: Chapman and Hall/CRC

[B33] Li W Yang Y Zipf’s law in importance of genes for cancer classification using microarray data J theor Biol 2002 219 4 539 551 10.1006/jtbi.2002.3145 12425984

[B34] Bornberg-Bauer E How are model protein structures distributed in sequence space? Biophys J 1997 73 5 2393 2403 10.1016/S0006-3495(97)78268-7 9370433 PMC1181141

[B35] Zipf G Human Behavior and the Principle of Least Effort 1949 Cambridge: Addison Wesley

[B36] Devroye L Limit laws for sums of functions of subtrees of random binary search trees SIAM J Comput 2003 32 152 171

[B37] Hwang HK Neininger R Phase change of limit laws in the quicksort recurrence under varying toll functions SIAM J Comput 2002 31 6 1687 1722 10.1137/S009753970138390X

[B38] Nebel ME Investigation of the Bernoulli model for RNA secondary structures Bull Math Biol 2004 66 5 925 964 10.1016/j.bulm.2003.08.015 15294413

[B39] Devroye L Universal limit laws for depths in random trees SIAM J Comput 1998 28 2 409 432 10.1137/S0097539795283954

